# Diversity of Trauma and Orthopaedic Trainees and Workplace Culture of Orthopaedic Training in the United Kingdom: Insights From the 2022 British Orthopaedic Trainee Association (BOTA) Census

**DOI:** 10.7759/cureus.75918

**Published:** 2024-12-17

**Authors:** Karen Chui, Kumar Kaushik Dash, Vasudev A Zaver, Adrian Andronic, James R Allen, James E Archer

**Affiliations:** 1 Trauma and Orthopaedics, British Orthopaedic Trainees Association, London, GBR

**Keywords:** bullying in healthcare, diversity and inclusion, resident well-being, surgeon burnout, workforce

## Abstract

The British Orthopaedic Trainee Association (BOTA) conducted its latest census in 2022 of its membership. The results of wellbeing, diversity, equity, inclusion and bullying are discussed here. This highlighted several key focuses for improvement. Firstly, further work is required to improve diversity within trauma and orthopaedics. Secondly, burnout amongst trainees is increasing, and we risk losing colleagues from the speciality without action. Finally, bullying and harassment are still witnessed within orthopaedics, and ways to address this are discussed.

## Introduction

In 2022, the British Orthopaedic Trainee Association (BOTA) [1] conducted the organisation’s census data collection for trauma and orthopaedic (T&O) trainees in the United Kingdom (UK). The 2022 census follows the 2019 study, which helped to inform the priorities for BOTA representation. The census surveys T&O trainees’ training experiences, workplace culture and the T&O community. It aims to collect the views and experiences of trainees to help inform the BOTA policies and provide evidence for advocacy on issues important to T&O trainees.

In this article, we highlight the census results related to the diversity of T&O trainees, trainees’ wellbeing and the experience of workplace culture.

In the UK, T&O is currently the largest surgical speciality with the highest number of consultants and speciality trainees [2]. However, T&O remains the least gender-diverse speciality, with female registrars making up 20.6% of the workforce and only 7.3% of consultants being female [3]. With regards to ethnic diversity, the British Orthopaedic Association (BOA) survey conducted in 2020 demonstrated that there were significant variations in the ethnicity of the workforce by grade, with 67% being White consultants and 30% being White medical students [4]. Increasing diversity in healthcare staff, including race, ethnicity, gender, cultural background and socioeconomic status, can have a considerable positive impact on patient outcomes. Providers that reflect the patients they care for can reduce healthcare disparities and improve patient outcomes [5,6].

The 2024 General Medical Council (GMC) National Training Survey [7] found that 40% of trainees feel burnt out because of work, which is a worryingly high rate. This is further compounded by a study by Jennings et al. [8], which showed from 2003 to 2017, orthopaedic surgeons experienced a high prevalence of burnout, depression and suicide; 28.2% of all surgeon suicides were found to be of orthopaedic surgeons, the highest prevalence of death by suicide amongst all surgical sub-specialities. We looked to assess factors that influence trainees' mental health and their access to support.

The 2015 ‘Undermining and Bullying in Surgical Training’ report published by the Association of Surgeons in Training [9] outlined the harmful nature of bullying in the workplace, not only from a staff wellbeing perspective but also from a patient safety perspective. Whilst bullying and harassment anecdotally appear to have reduced over the years, this report highlights that it has still not been eradicated. We aimed to assess trainees' experience of bullying and harassment and attitudes towards reporting incidents and the common perpetrators.

Therefore, these key areas form the focus of this article and lead to recommendations on how to make improvements.

## Materials and methods

The census survey questions were created by the BOTA committee and aimed to assess a wide range of concerns and collect a comprehensive overview of T&O training in the UK. Some questions were repeated from the 2019 census, whilst others were adapted to reflect changes between 2019 and 2022. One hundred and one questions were selected and are displayed in the Appendices.

The census was completed online using Survey Monkey (SurveyMonkey, San Mateo, CA, USA) [10]. The survey was sent to all of the BOTA membership by email as well as being advertised through social media. The survey was open for completion for a period of three months. Completion of the census was incentivised with a prize draw for all respondents who completed the census survey. The prize offered was coverage of the Intercollegiate Surgical Curriculum Programme fees for one year (£260).

All T&O trainees in the UK who are members of the BOA/BOTA were eligible to complete the survey. This was confirmed by the entry of a national training number (NTN) or BOA/BOTA membership number. Exclusion criteria were completions without a listed NTN or BOA/BOTA number. All responses were anonymised. No institutional review board approval was sought for this survey.

Data was summarised in graphical and tabular format by Survey Monkey. The results were then analysed by the BOTA committee. Analysis was performed by comparing results to the 2019 results and comparing them to the already published results in the literature to identify trends and differences.

## Results

The 2022 BOTA census had 549 total responses compared to 843 responses in 2019. The results are broken down into key areas below.

Diversity

In 2022, of the 450 respondents, 128 (28.4%) identified as cisgender women, 295 (65.6%) as cisgender men, 16 (3.56%) chose ‘prefer not to say’, one (0.2%) as a transgender man, one (0.2%) as a genderqueer and one (0.2%) as an agender.

With regards to ethnic diversity, of the 450 respondents, 262 (58%) identified as White individuals, 65 (14.4%) as Asian Indian, Pakistani, or Bangladeshi individuals, 22 (4.9%) as Asian Chinese individuals, eight (1.7%) as Black individuals and 23 (5.1%) as Arab individuals. This is shown in more detail in Table 1.

**Table 1:** Ethnicity of respondents in 2019 and 2022

Ethnicity	2019		2022	
	n	%	n	%
White - British/English/Northern Irish/Scottish/Welsh	333	51.79%	253	56.22%
White - Irish	22	3.42%	9	2.00%
White - Gypsy or Irish Traveller	0	0%	0	0%
Mixed/Multiple - White and Black Caribbean	2	0.31%	1	0.22%
Mixed/Multiple - White and Black African	2	0.31%	2	0.44%
Mixed/Multiple - White and Asian	11	1.71%	8	1.78%
Asian/Asian British - Indian	81	12.60%	42	9.33%
Asian/Asian British - Pakistani	23	3.58%	21	4.67%
Asian/Asian British - Bangladeshi	5	0.78%	2	0.44%
Asian/Asian British - Chinese	27	4.20%	22	4.89%
Black/African/Caribbean/Black British - African	17	2.64%	6	1.33%
Black/African/Caribbean/Black British - Caribbean	0	0%	2	0.44%
Arab	33	5.13%	23	5.11%
Prefer not to say	45	7.00%	22	4.89%
Other (please specify)	42	6.53%	37	8.22%
Total	643	-	450	-

Burnout

The 2019 and 2022 BOTA census included the Copenhagen Burnout Inventory (CBI) [11] to assess work-related burnout. The results of the CBI responses are presented in Table 2 for the 2019 census and Table 3 for the 2022 census.

**Table 2:** Work-related burnout in the 2019 census Total average score (2019 census) = 45.28 (standard deviation 25.71) Respondents (n) = 636

Copenhagen Burnout Inventory – 2019 census	Always % (n) (score 100)	Often % (n) (score 75)	Sometimes % (n) (score 50)	Seldom % (n) (score 25)	Never % (n) (score 0)
Is your work emotionally exhausting?	1.10% (7)	16.35% (104)	45.91% (292)	27.83% (177)	8.81% (56)
Do you feel burnt out because of your work?	1.57% (10)	14.96% (95)	44.09% (280)	27.40% (174)	11.97% (76)
Does your work frustrate you?	2.53% (16)	23.22% (147)	49.92% (316)	17.85% (113)	6.48% (41)
Do you feel worn out at the end of the working day?	7.26% (46)	34.86% (221)	40.38% (256)	13.72% (87)	3.79% (24)
Are you exhausted in the morning at the thought of another day at work?	2.68% (17)	16.85% (107)	33.70% (214)	30.87% (196)	15.91% (101)
Do you feel that every working hour is tiring for you?	0.79% (5)	5.03% (32)	19.03% (121)	39.47% (251)	35.69% (227)
Do you have enough energy for family and friends during your leisure time?	9.28% (59)	45.13% (287)	30.97% (197)	12.74% (81)	1.89% (12)

**Table 3:** Work-related burnout in the 2022 census Total average score (2022 census) = 49.85 (standard deviation 25.30) Respondents (n) = 440

Copenhagen Burnout Inventory – 2022 census	Always % (n) (score 100)	Often % (n) (score 75)	Sometimes % (n) (score 50)	Seldom % (n) (score 25)	Never % (n) (score 0)
Is your work emotionally exhausting?	2.95 % (13)	23.13 % (102)	48.30 % (213)	18.82 % (83)	6.80 % (30)
Do you feel burnt out because of your work?	5.02 % (22)	23.06% (101)	44.52% (195)	19.41 % (85)	7.99% (35)
Does your work frustrate you?	3.89% (17)	28.60% (125)	49.20% (215)	14.65% (64)	3.66% (16)
Do you feel worn out at the end of the working day?	14.32% (63)	38.86% (171)	35.45% (156)	9.09% (40)	2.27% (10)
Are you exhausted in the morning at the thought of another day at work?	6.16 % (27)	22.60% (99)	36.30% (159)	25.80% (113)	9.13% (40)
Do you feel that every working hour is tiring for you?	1.82 % (8)	8.41 % (37)	27.05% (119)	41.36% (182)	21.36% (94)
Do you have enough energy for family and friends during your leisure time?	6.36 % (28)	33.64% (148)	33.41% (147)	21.36% (94)	5.23 % (23)

Mental health

In the 2019 census, 49.8% (317 of 637) of trainees experienced episodes of stress or anxiety during their training that have repeatedly affected their quality of life. The majority of this was related to their work, with only 8% (51 of 637) reporting it was unrelated to work. In 2022, this increased significantly to 56.1% of trainees answering "yes," it was related to their work, with only 11.5% (51 of 442) reporting it was unrelated to work. This is demonstrated in Table 4 below.

**Table 4:** Comparison of responses to a survey question between 2019 and 2022

Question	Responses	2019	2022
Have you experienced any episodes of stress or anxiety during your specialist/higher surgical training that have repeatedly affected your quality of life?	Yes – related to my work	41.8% (266)	56.1% (248)
	Yes – unrelated to my work	8% (51)	11.5% (51)
	No	50.2% (320)	32.4% (143)
		N = 637	N = 442

Reasons for low morale varied, as demonstrated in Table 5 below. Common factors such as operating experiences (30.3%, 134 of 442), rota gaps (22.4%, 99 of 442), workload (21.3%, 94 of 442), geographic location of the hospital (20.4%, 90 of 442), and personal factors outside of work (20.4%, 90 of 442) were identified.

**Table 5:** Factors that affected morale negatively from the 2022 census ARCP: annual review of competence progression, TPD: training programme director

Factors that influenced morale negatively	
Operative experiences	30.3% (134)
Rota gaps	22.4% (99)
Workload	21.3% (94)
Geographical location of the hospital	20.4% (90)
Personal factors outside of work	20.4% (90)
Due to the rota (excluding gaps)	19.9% (88)
On-call intensity	17.7% (78)
Other	14.5% (64)
Child care/carer duties	11.1% (49)
ARCP	10.9% (48)
Colleagues	10% (44)
Leave availability	8.1% (36)
Other consultants in the department	7.7% (34)
My consultant trainer	5.7% (25)
My TPD	3.2% (14)
None of the above	8.8% (39)
	N = 442

In 2022, 10.5% (47 of 441) of trainees said they have a physical or mental health condition lasting or expected to last for 12 months or more; 47.7% (21 of 44) of trainees with conditions said that their workplace offers appropriate work-based adaptations to enable them to fulfil clinical duties to their full potential; 13.15% (58 of 441) of trainees have accessed mental health support services in the 12 months prior to the census, with only 29% (128 of 441) of respondents saying this is provided by their workplace or training region; 78.9% (348 of 441) of trainees think the support should be provided by their workplace or training region. The prevalence of suicidal thoughts due to work is also rising from 2% (13 of 636) in 2019 to 4.1% (18 of 441) in 2022.

Bullying and harassment

In the four weeks prior to completing the survey, 17.7% (78 of 441) of respondents reported direct experience of bullying, and 27.3% (120 of 439) had witnessed bullying; 77.2% (61 of 69) of those affected by bullying did not report it.

In the four weeks prior to completing the survey, 9.4% (41 of 438) had felt harassed at work, and 13.5% (59 of 437) had witnessed harassment at work. A further 33.9% (148 of 437) felt undermined, and 30.5% (133 of 436) witnessed others being undermined.

Harassment was reported to be related to sex (37.2%, 16 of 43), race (21%, 9 of 43), and religion (14%, 6 of 43), whilst 18.6% (8 of 43) preferred not to say. Unfortunately, a similar rate of non-reporting was seen, with 79.1% (34 of 43) of those affected by harassment not reporting it.

Tables 6-7 below demonstrate the identified perpetrators of bullying and harassment, respectively.

**Table 6:** Perpetrators of bullying in the 2022 census T&O: trauma and orthopaedics, AHPs: allied health professions, ODPs: operating department practitioners, SpR: specialist registrar, TPD: training programme director, FT/CT: foundation trainee/core trainee Respondents (n) = 79

Perpetrators of bullying	n	%
Consultant (T&O)	44	55.70%
Hospital manager	16	20.25%
Nurse/scrub nurse	12	15.19%
Consultant (anaesthetist)	10	12.66%
AHPs theatre	10	12.66%
Consultant (other)	7	8.86%
ODPs	7	8.86%
SpR	5	6.33%
TPD	5	6.33%
Patient	4	5.06%
Fellow	3	3.80%
Theatre coordinator	3	3.80%
Patient relatives	1	1.27%
Other (please specify)	1	1.27%
FT/CT doctor	0	0.00%
Medical student	0	0.00%
Secretary	0	0.00%

**Table 7:** Perpetrators of harassment in the 2022 census T&O: trauma and orthopaedics, AHPs: allied health professions, ODPs: operating department practitioners, SpR: specialist registrar, TPD: training programme director, FT/CT: foundation trainee/core trainee Respondents (n) = 43

Perpetrators of harassment	n	%
Consultant (T&O)	18	41.86%
Other (please specify)	7	16.28%
Nurse/scrub nurse	6	13.95%
Patient	6	13.95%
Consultant (other)	4	9.30%
Consultant (anaesthetist)	3	6.98%
Fellow	3	6.98%
Hospital manager	3	6.98%
AHPs theatre	2	4.65%
SpR	1	2.33%
TPD	1	2.33%
ODPs	1	2.33%
Patient relatives	1	2.33%
FT/CT doctor	0	0.00%
Medical student	0	0.00%
Secretary	0	0.00%
Theatre coordinator	0	0.00%

## Discussion

The need to increase diversity in the workforce

As previously discussed, the number of female consultants working in T&O is low. However, these numbers are starting to change. In the 2019 BOTA census, 23.3% of respondents identified as female. In the 2022 census, this has increased further to 28.4%, showing a gradual positive trend. Changing the gender balance in T&O will not be a quick process, with the training pathway often taking up to ten years to complete. However, the change in our census is a positive indicator that we see changes in the future.

Recent studies have suggested positive impacts of female surgeon sex on patient outcomes. Wallis et al. [12] conducted a retrospective cohort study of the effect of surgeon sex on the rate of adverse postoperative events in patients undergoing 25 common elective or emergency surgeries between 2007 and 2019 in Canada. The study included 151,054 patients treated by a female surgeon and 1,014,657 patients treated by a male surgeon. After adjusting for patient, procedure, surgeon, anaesthetist and hospital characteristics, the study found that female surgeons had lower rates of death at 90 days and one year after surgery. The potential positive impact of the gender of the surgeon on patient outcomes is emerging and warrants further research.

Interestingly, over 32.5% of respondents reported feeling comfortable with their team, including their seniors, knowing their sexual orientation. However, 50% of the respondents reported not being open about their sexual orientation.

Increasing LGBTQ+ representation, allyship awareness and bystander training are different strategies that can be used to create a safer environment and a more accepting culture for LGBTQ+ surgeons in T&O. This is an area actively being advocated for by both the BOA and BOTA with the recruitment of culture and diversity champions to help drive these improvements.

Ethnicity

With regard to ethnicity, there has not been a significant change from 2019 to 2022. The advantages of increased workforce diversity, including improved organisation performance, innovation, profitability and staff wellbeing, are well reported in many industries. McKinsey’s 2023 ‘Diversity Matters’ report demonstrated that companies in the top quartile for gender and ethnic diversity are 39% more likely to outperform peers [13]. In the healthcare industry, multiple studies have demonstrated the benefits of a more diverse medical workforce on patient outcomes, patient satisfaction, staff wellbeing and staff performance [5,14,15].

Educating healthcare providers of diverse cultural backgrounds and beliefs of patients can lead to more culturally competent care. Cultural competence in healthcare refers to the ability of healthcare providers and organisations to understand, respect and effectively respond to the cultural and linguistic needs of patients from diverse backgrounds [16]. Improved cultural competence can lead to improved patient communication and adherence to care, reduce barriers to accessing health and has been shown to improve patient outcomes and reduce healthcare disparities [17]. A systematic review by Truong et al. found moderate evidence that interventions to improve cultural competence led to improvements in provider outcomes, healthcare access and utilisation outcomes [18]. Other studies have demonstrated that patients’ perceived cultural competency of their physician increases patient engagement in cancer screening [19].

Another benefit of increasing diversity in the surgical workforce is improved patient satisfaction. Patients from under-represented groups report higher patient satisfaction and better patient-provider communication when receiving care from physicians of minority backgrounds. Higher patient satisfaction with care can lead to increased patient compliance with care and participation in treatments [20,21].

Increased diversity in the workplace can promote a sense of inclusion and belonging, leading to increased staff wellbeing and building a positive work culture [22]. Ensuring positive staff wellbeing is important as it is associated with patient safety. A systematic review by Hall et al. [23] reported that healthcare staff's poor wellbeing was associated with poorer patient safety and increased errors. Staff burnout was found to be associated with more errors and lower patient safety grades.

It is for this myriad of reasons that BOTA wants to advocate for and encourage trainees from a more diverse background. The culture and diversity champions are expected to be role models to inspire trainees from more diverse backgrounds to enter orthopaedic training.

Burnout

Burnout is being increasingly recognised amongst healthcare professionals worldwide. Its definition has evolved over the years, although the key concept remains largely the same: a state of physical, emotional and mental exhaustion that results from long-term involvement in work situations that are emotionally demanding [24].

Notably, the burnout scores from the census were high in 2019, and this trend will continue in 2022. For reference, in the PUMA baseline prospective study, the average score for work-related burnout across employees in various professions in human service work was 33 (SD 17.7) [25], and the score for hospital doctors was 39.8. Worryingly, the average score has risen from 2019 to 2022. The average score in 2019 was 45.28 (SD 25.71), and in 2022, it was 49.85 (SD 25.30). This is clearly far higher than the PUMA baseline study and inevitably led to eighteen respondents reporting that they had to take time off work due to work-related burnout. The actual incidence of burnout is likely to be higher, as burnout is associated with disengagement and exhaustion, which may have been a barrier to participating in the census.

Trainees continue to face high rates of burnout, which can negatively affect the quality of care for patients and doctors’ wellbeing. NHS employers have published guidance for NHS trusts to take action to address burnout [26]. It includes practical tips such as ensuring optimum staffing levels and overcoming the stigma of mental health conversations at work. Employers, trusts, and deaneries must intervene to improve the wellbeing of UK orthopaedic trainees and prevent burnout. BOTA recognises these concerns, and the Top 5 Priorities for Trainees released by BOTA aim to address many of these with the aim of improving wellbeing.

Mental health

The mental health of trainees is also showing a concerning trend. There are many trainees with mental health conditions; however, the number who report accessing mental health services is higher. More than two-thirds of our trainees do not have access to these services. There has also been a worrying increase in the rates of suicidal thoughts related to work.

Many of the common reasons for low morale are areas that can be proactively improved within individual departments, including operating experiences, rota gaps, and workload. Improvements in these areas will have a positive impact on the mental health of trainees.

Especially with evidence that levels of suicidal thoughts are rising, mental health support services are essential for the wellbeing of orthopaedic trainees, and employers should continue to fund this important resource.

BOTA has developed a range of guidance and support on wellbeing and associated issues. These are all available on the BOTA website and aim to help support trainees and direct them to further sources of support.

Bullying and harassment

The 2022 census provided valuable insight into bullying and harassment in T&O. As shown in Table 6, consultant T&O surgeons were the most common perpetrators of bullying. Similarly, they were the most common perpetrators of harassment, too.

Many of those affected by bullying and harassment had not reported it. The reasons individuals choose not to report are multifactorial and are often due to a lack of confidence in accountable organisations dealing with the complaint and fear of consequences.

Our census highlights that bullying and harassment are still prevalent in T&O surgery and complements the results of the 2024 GMC National Training Survey [7], which found that unprofessional and discriminatory behaviours continue to take place in the workplace, and factors such as gender, ethnicity, religion and sexual orientation affect a trainees’ experience.

Of particular concern is that the majority of harassment cases were related to sex. This disappointingly echoes the findings of the recent landmark reports on sexual harassment in surgery by the Working Party on Sexual Misconduct in Surgery [27]. The 2023 NHS staff survey [28] demonstrated that 9% of staff reported unwanted behaviour of a sexual nature from patients, relatives and the public, and 3.8% from colleagues. Ethnic minorities face more discrimination from patients, managers and colleagues (28.6%) than White colleagues (24.7%) and should be addressed as a matter of priority.

Bullying, harassment and discriminatory behaviours can negatively impact the individual, teamwork, communication and, ultimately, patient care. The Royal College of Surgeons England (RCS Eng) Kennedy report provided clear parameters of behaviours and attitudes we should expect within our workforce [29]. The RCS Eng ‘Managing Disruptive Behaviours in Surgery’ report [30] explains the negative impact of bullying and harassment on staff and patient care and provides guidance on how to challenge these behaviours. The GMC runs professional behaviours and patient safety workshops, which provide training on how to challenge unprofessional behaviours and maintain effective working relationships. These reports and initiatives are a welcome change to support the efforts going into improving the workplace culture in surgery.

Limitations

Whilst the number of census completions was high, this still represents a limitation of the study as to be truly representative of the workforce, and we would need 100% completion. However, the results align with the previous census results and other work by the BOA and, therefore, are likely to be a good representation of the trainees in the UK.

Not all respondents answered all of the questions in the survey, meaning that the number of respondents for different questions is varied. This is probably due to survey fatigue and attempts to complete the survey as quickly as possible. However, the census was purposefully designed not to require answers for all subjects, as some individuals may not wish to disclose this information. For future iterations of the census, this may need to be reviewed to ensure a good response rate.

Our census also utilised categorical answers for the vast majority of the questions to ensure the process was quick and easy for trainees to complete. However, this may lead to a reduction in the complexity and variety of answers offered by more open-ended answers.

## Conclusions

Increasing the diversity of our workforce is crucial to creating a more inclusive environment, improving staff well-being and performance and improving patient satisfaction and outcomes. Strategies to improve diversity and inclusion require initiatives at multiple levels, from medical school to post-graduate training and include outreach programs, mentorship, visible role models, diverse leadership, allyship, diversity, equity, and inclusion training, research, unconscious bias training, flexible working patterns and collaborative effort from all members of the speciality. BOTA strongly advocates for this, and as the census results demonstrate, we are already starting to see increasing numbers of female trainees, and we are keen to keep this positive trend of improving diversity within the T&O workforce.

As highlighted by the results of this survey, staff burnout, mental health, staff wellbeing and incidence of bullying and harassment are all key concerns for trainees, and co-ordinated action as a speciality is essential to address this. BOTA works for trainees on a national level on a wide range of committees. The results of this census have helped to form the BOTA Top 5 Priorities for Trainees 2024 and will continue to help drive our efforts for improvements in the future.

## Appendices

Questions included in the 2022 BOTA survey

1.     What is your training grade?

2.     What is your training region?

3.     Have you completed FRCS (T&O) examinations (parts 1 and 2)?

4.     Are you working less than full-time (LTFT)?

5.     What category of activities are you doing when not clinical work?

a.     Research

b.     Further education (PhD, MD)

c.     Personal time

d.     Carer duties

e.     Child care

f.      Health reasons

g.     Business reasons

h.     Academic teaching/lecturer

i.      Leadership/management role

j.      Other (please specify)

6.     Are you currently out of programme (OOP)?

7.     What are you doing with your OOP time?

a.     Research

b.     Career break

c.     Seeking a focused clinical experience

d.     Parental leave

e.     Non-clinical fellowship (leadership/management/education)

f.      Clinical fellowship

g.     Other (please specify)

8.     Please indicate how this time out of training is registered with your deanery

9.     What is your gender?

10.   What is your relationship/marital status?

11.    Which sexuality best describes yours?

12.   Choose one option that best describes your ethnic group or background

13.   Did you attend a fee-paying high school/secondary education?

14.   Did you attend a medical school on scholarship or with financial aid?

15.   Do you have dependents/children?

16.   Are you open about your sexual orientation with colleagues at work?

17.    Do you have any physical or mental health conditions or illnesses lasting or expected to last for 12 months or more?

18.   Do any of your conditions or illnesses reduce your ability to carry out day-to-day activities?

19.   Does your T&O workplace offer appropriate work-based adaptations to enable you to fulfil clinical duties to your full potential?

20.   Do any of these conditions or illnesses affect you in any of the following areas?

21.   Do you have a rotation-wide ‘lead employer’ (e.g., one that streamlines and coordinates occupational health and payroll between your hospital/trust placements)?

22.   Have you considered leaving T&O over the last 24 months?

23.   During the last four weeks, to what extent do you agree or disagree with the statement ‘I enjoy my work’?

24.   Do you feel supported by your TPD?

25.   Have any of the following factors influenced your morale negatively in the last four weeks? (Max 3 choices)

a.     Colleagues

b.     My consultant trainer

c.     Rota (excluding rota gaps)

d.     Rota gaps

e.     Workload

f.      Operating experiences

g.     On-call intensity

h.     Leave availability

i.      My TPD

j.      Other consultants in the department

k.     Personal factors outside work

l.      ARCP

m.    Geographic location of hospital

n.     Child care/Carer duties

o.     None of the above

p.     Other (please specify)

26.   Have you experienced any episodes of stress or anxiety during your specialist/higher surgical training that have repeatedly affected your quality of life?

27.    Have you experienced issues related to your personal wellbeing directly related to COVID-19 pandemic pressures on training?

28.    Burnout

a.      Is your work emotionally exhausting? 

b.      Do you feel burnt out because of your work? 

c.      Does your work frustrate you? 

d.      Do you feel worn out at the end of the working day? 

e.      Are you exhausted in the morning at the thought of another day at work? 

f.       Do you feel that every working hour is tiring for you? 

g.      Do you have enough energy for family and friends during leisure time?

29.    Have you had time off work for burnout?

30.    Have you experienced any suicidal thoughts?

31.    Have you accessed mental health support services in the past 12 months?

32.    Does your workplace or training region provide mental health or psychological support?

33.    Do you think mental health or psychological support should be provided by your workplace or training region?

34.    In the last 4 weeks, have you felt bullied at work?

35.    Who was the perpetrator?

36.    Did you report this to anyone?

37.    If you have been bullied, please provide details here. If you choose not to report it, please explain why.

38.    In the last 4 weeks, have you witnessed others in your orthopaedic team being bullied?

39.    In the last 4 weeks, have you felt harassed at work?

40.    My harassment was related to...

41.     Who was the perpetrator?

42.    Did you report this to anyone?

43.    If you have been harassed, please provide details here. If you choose not to report it, please explain why.

44.    In the last 4 weeks, have you witnessed others in your orthopaedic team being harassed?

45.    In the last 4 weeks, have you felt undermined at work?

46.    Who was the perpetrator?

47.     Did you report this to anyone?

48.    If you have been undermined, please provide details here. If you choose not to report it, please explain why.

49.    In the last 4 weeks, have you witnessed others in your orthopaedic team being undermined?

50.    How well does your current post meet your training needs?

51.    On your current rota, how many (SPR) rota gaps are there?

52.    In the last 4 weeks, how often have you been expected to cover an extra outpatient clinic and/or theatre session because of sickness, leave, rota gap, or service pressure?

53.    In the last 4 weeks, how often have you been expected to cover an on-call shift because of sickness, leave, rota gap, or service pressure?

54.    Are you currently in a clinical placement?

55.    How satisfied are you that your operative exposure is adequate to be on track to meet the requirements of the CCT indicative numbers?

56.    What is the primary barrier to you being the first/primary surgeon (i.e. STS/STU/P)? (Select 3 max)

57.    What sort of professional support should be available to surgical trainers?

58.    When did you decide on a career in T&O surgery?

59.    If your postgraduate training were to be reduced, which of the following would best match your opinion?

60.    Does your region promote or encourage working in low- and middle-income countries (LMICs) as a trainee?

61.    Have you worked in a LMIC as a doctor?

62.    If you have NOT worked in a LMIC, what are your reasons?

63.    How much time have you spent working in a LMIC as a doctor?

64.    Did your time working in LMIC count towards your training, either in full or part (including study leave)?

65.    Was your overseas work paid or unpaid?

66.    Was your engagement in LMIC work principally...

67.     Would work in an LMIC for any length of time appeal to you if it counted towards your CCT requirements?

68.    Would an 'overseas orthopaedics' placement appeal to you if incorporated into your training programme?

69.    Have you trained in the private sector as part of your contractual hours? (Not including voluntary assistance with private lists.)

70.    In the private sector, what clinical areas were you working in? (Select options that apply)

71.    In the private sector, how would you describe the quality of your training experience?

72.    In the private sector, how would you compare your private training experiences to training in the NHS?

73.    Do you have access to simulation training as part of your deanery?

74.    Please rate the quality of the simulation training

75.    Have you had any formal training in computer-assisted/robotic orthopaedic surgery?

76.    Please rate the quality of the computer-assisted/robotic training

77.    Are you aware of the requirements (IRMER) for Ionising radiation in your workplace prior to starting your current job?

78.    How often do you use ionising radiation (image intensifier - standard or mini)?

79.    How often do you wear the following radiation protection equipment for all cases involving the use of the image intensifier? Please choose the most appropriate response for each.

80.    'I feel adequately trained in ionising radiation safety knowledge and legislation'. Please select from the responses below.

81.    'My current trust provides adequate radiation protective equipment for me when in the operating theatre'. Please select from the responses below.

82.    How would you rate the quality of the ionising radiation protection equipment at your workplace?

83.    How have the new CCT requirements (published August 2021) affected you?

84.    Which indicative procedure have you found the most difficult to attain?

85.    How many MCRs did you have in your last 6-month placement?

86.    Who initiated the MCR process?

87.    Did these MCRs include a formal feedback session with your CS/AES?

88.    How useful was this feedback

89.    Have you sat the FRCS (Tr&Orth) examination?

90.    How satisfied were you with the location of the FRCS (Tr&Orth) part 1 exam?

91.    Did you encounter any disruption during the part 1 exam?

92.    Did you undergo ST3 recruitment via a virtual interview process?

93.    Please rate your overall experience on the day of your virtual interview

94.    Did you have any audio/visual difficulties?

95.    If you had AV difficulties, did this impact your overall performance?

96.    Did you have any internet/connectivity difficulties?

97.    If you had internet/connectivity difficulties, did this impact your overall performance?

98.    Would you recommend future interview processes via remote/non- F2F methods?

99.    Do you feel face-to-face interview stations represent real practice in the following domains:

a.      Technical skills

b.      Prioritisation

c.      Communication

100.   Have you developed an interest in a particular sub-speciality?

101.    Do you wish to be an academic orthopaedic surgeon (e.g., a university appointment with a split clinical/academic workload)?

102.   Are you planning to undertake a post-CCT fellowship?

103.   What is your preferred total duration for a fellowship(s)?

104.   What influences your decision to complete a fellowship(s)?

105.   When choosing your fellowship, apart from sub-speciality, which additional factors are most important?

106.   At present, do you feel able to afford the overall cost of training? (e.g. professional fees, educational material, commuting, exam fees, conferences/courses)

107.   Has the new ‘cost of living crises’ personally affected your training participation?

108.   Compared to this time last year, do you feel financially...

109.   How concerned are you about the current cost of living crisis?

110.   Which of the following, if any, are you concerned about as a result of the current cost of living crisis? Please select all that apply.

a.      Covering travel costs (to and from work)

b.      Covering paid courses/conferences

c.      Fellowship implications

d.      Saving for future life

e.      Covering mortgage payments

f.       Incurring debts

g.      Housing/rent

h.      Education/childcare costs for dependents

i.       Other (please specify)

111.   How do you plan to mitigate increased costs of living?

a.      Reduce the number of paid courses

b.      Reduce attendance at paid educational meetings

c.      Increase LTFT hours (e.g. 50% to 80%)

d.      Locums

e.      Apply for additional personal loan

f.       Use of a food bank

g.      Reduce social expenses

h.      Move property

i.       Other (please specify)

112.   Are financial worries having an impact on your mental health?

113.   How useful is BOTA to you as a trainee?

114.   Can you name your BOTA Regional Representative (previously known as a Linkman)?

115.   Have you ever contacted your Regional Representative regarding a training issue?

116.   Do you feel that your Regional Representative has been elected fairly and democratically (i.e. with a vote amongst your region’s trainees)?

117.   What most stops you from standing for election and joining the BOTA committee?

118.   In what way could BOTA better represent or serve you and other trainees?

119.   I think full BOTA membership should be open to all orthopaedic interested junior doctors (i.e. non-training scheme doctors).

120.   Do you think BOTA should provide funding for trainees or medical students to run courses?

121.   What areas do you think should be a priority with regard to representation for BOTA over the next 24 months?
